# Severity and Outcomes of Dengue in Hospitalized Jamaican Children in 2018–2019 During an Epidemic Surge in the Americas

**DOI:** 10.3389/fmed.2022.889998

**Published:** 2022-06-21

**Authors:** Aileen May Lue, Michelle-Ann Elizabeth Hue Richards-Dawson, Georgiana Marie Gordon-Strachan, Syed Matthew Kodilinye, Jacqueline Anne Theresa Dunkley-Thompson, Tracia Dahlia James-Powell, Curtis Alphonso Pryce, Chadwic De'sean Mears, Joshua James Anzinger, Karen Webster-Kerr, Celia Dana Claire Christie

**Affiliations:** ^1^Bustamante Hospital for Children, Kingston, Jamaica; ^2^Pediatric Residency Program, University of the West Indies, Kingston, Jamaica; ^3^Caribbean Institute of Health Research, University of the West Indies, Kingston, Jamaica; ^4^ZIKAction Research Consortium, Fondazione Penta, University of Padova, Padova, Italy; ^5^Department of Child and Adolescent Health, University Hospital of the West Indies, Kingston, Jamaica; ^6^Department of Pediatrics, Mandeville Regional Hospital, Mandeville, Jamaica; ^7^Department of Pediatrics, Spanish Town Hospital, Spanish Town, Jamaica; ^8^Department of Pediatrics, May Pen Hospital, May Pen, Jamaica; ^9^Department of Microbiology, University of the West Indies, Kingston, Jamaica; ^10^Global Virus Network, Baltimore, MD, United States; ^11^National Epidemiology Unit, Ministry of Health and Wellness, Kingston, Jamaica; ^12^Department of Child and Adolescent Health, University of the West Indies, Kingston, Jamaica

**Keywords:** dengue, severe dengue, dengue serotype, antibody-dependent enhancement, children, immunity, Jamaica

## Abstract

**Objective:**

In 2019, dengue was among the “top-ten threats to global health,” with 3.1 million cases reported from the Americas, the highest ever. Simultaneously, Jamaica reported its largest dengue outbreak in 40 years, following Chikungunya and Zika virus epidemics, in 2014 and 2016–2017, respectively. We describe dengue in children admitted to five hospitals in Jamaica during August 2018 through September 2019.

**Methods:**

Hospitalized children and adolescents aged 0 to 15 years with dengue were managed using PAHO/WHO criteria. Data were extracted from questionnaires, entered into a dataset on Microsoft Excel version 2016, exported to SPSS version 20 and analyzed. Groups were compared using Student's *t*-test for normally distributed parametric data. Chi-square analysis, or Fisher's exact test was used for categorical variables. A *p-*value < 0.05 was considered statistically significant.

**Results:**

There were 339 children, 245 (72.3%) aged 1–10 years, males:females 1:1. Classification was “dengue without warning signs” 53 (15.3%), “dengue with warning signs” 218 (64.3%) and “severe dengue” 68 (20%). Co-morbidities were reported in 88 (26%). Hemoglobin SC disease was associated with severe dengue with hemorrhage (*p* = 0.005). Organ-system involvement occurred in 334 (98.5%) including gastrointestinal 317 (93.5%), hematologic 311 (91.7%) and musculoskeletal 180 (53.1%). Thirty-nine (11.5%) had 5–7 organ-systems involved. Metabolomics emphasized increased hepatic transaminases 245 (72.3%), lactate dehydrogenase 164 (48.4%) and creatine phosphokinase 84 (24.8%) approaching the high thousands (121,560 u/L), both were markers for severe disease (*p* < 0.002). Thirteen (3.8%) received intensive care. Dengue was laboratory-confirmed in 220 (78.9%): NS1 antigen-positive (218); RT-PCR-positive (23), with an overlap of NS1 antigen and RT-PCR positive (21); DENV-3 serotype (20). Seventeen (5%) died, 16 (94.1%) had severe dengue and 11 (64.7%) succumbed within 24 to 48 h of admission despite resuscitation and transfusion of blood products.

**Conclusion:**

Severe dengue with increased attributable mortality occurred in hospitalized children after Jamaica's maiden Zika epidemic.

## Introduction

Dengue was designated one of the “top ten threats to global health” in 2019 by the World Health Organization (1). It is a leading cause of death and illness among persons with arbovirus infections (2). Over half the world's population is at risk, over 390 million cases are reported annually and 96 million present clinically with varying disease severity (3). Dengue is caused by the arthropod-borne flavivirus with four dengue virus (DENV) serotypes DENV-1, 2, 3 and 4 (1). Transmission is mainly by the *Aedes aegypti* mosquito (4). In 2009, the WHO re-classified dengue infection: “dengue without warning signs,” “dengue with warning signs,” and “severe dengue with and without hemorrhage” (5). In Latin America and the Caribbean, over 3.1 million cases were reported in 2019, the highest ever historically, with all four serotypes (1–4) co-circulating in Brazil, Guatemala and Mexico (1, 6). The proportion of severe dengue, 0.9%, exceeded reports in the preceding 4 years (6). Children typically have the most severe presentations of dengue and the highest attributable-morbidity and mortality (7).

In Jamaica, an upper middle-developing Caribbean island-nation, with population 2.97 million, national surveillance data are available from 1977 for dengue epidemics, where sero-prevalence rates approach 99–100% in pregnancy, which is a readily-accessible representative Jamaican subpopulation (8). In 2014, an epidemic with several thousands of suspected cases of chikungunya virus (CHIKV) infections were reported with an 83.6% seropositivity (9–12). In 2016, Jamaica experienced its maiden Zika virus (ZIKV) epidemic, when all three arboviruses co-circulated endemically (13–16). In Cuba, two *Ae aegypti* mosquito populations became infected with and transmitted DENV-1, ZIKV and CHIKV together, albeit at low rates (17).

Dengue outbreaks have increased in frequency and intensity over 40 years of active dengue surveillance in Jamaica, with increased intensity, severity and reported cases in recent years. According to the national surveillance data, there were 5,461 dengue-reported cases in 2007 and 5,903 in 2012 (16). Jamaica experienced an upsurge in cases late 2018 with an epidemic declared on January 3, 2019 (18). In Jamaica, during 2012, among 134 children who were admitted with dengue to the academic referral center, the University Hospital of the West Indies (UHWI), 10.45% were severe with a case fatality rate (CFR) of 3.73% (19). The circulating serotype was DENV-3 (13). In the 2018-2019 dengue epidemic, children were also significantly affected (6, 16). There were 10,411 suspected and confirmed total dengue cases in adults and children in the 2018–2019 epidemic of which 22% required hospitalization (16). Among adults and children, there were 86 suspected and confirmed dengue-related deaths island-wide in 2018–2019 with an overall national case fatality rate (CFR) for the combined ambulatory and hospitalized cases who died of 0.83%. This has increased from 0.39% in 2012 and 0.46% in 2007 (16). Jamaica's dengue mortality rates therefore exceed PAHO's dengue-attributable mortality threshold of 0.05% for the Americas (20).

There is a paucity of reports describing the clinical severity of dengue in children in the aftermath of the ZIKV “epidemic of international concern” that was declared in the Americas. Initial reports by the pediatricians and national surveillance of high attributable-morbidity and mortality in children in the 2018–2019 dengue epidemic in Jamaica, were of major importance. Information obtained about severe dengue in this setting can be used to further inform other regions and further elucidate the patho-physiology of “severe dengue” in similar populations of children who are hospitalized with dengue fever.

The primary objective of the study was to evaluate and describe clinical and laboratory-confirmed cases of dengue in hospitalized Jamaican children ages 0–15 years at five hospitals during the 2018–2019 epidemic using WHO diagnostic criteria. Additionally, we identified the associated clinical factors and laboratory markers of severe disease.

## Materials and Methods

This ambispective cohort study was conducted for suspected and confirmed dengue cases identified before February 2019 and prospectively thereafter. Cases were included and enrolled from August 2018 to September 2019 during the epidemic. DENV, CHIKV and ZIKV infections are all reportable Class 1 Notifiable Diseases to the Jamaican Ministry of Health and Wellness (MoHW) and immediately, within 24 h of suspicion. Five hospitals across Jamaica, serving about half the island's pediatric population were included: Bustamante Hospital for Children, providing services for children aged 0–11 years; University Hospital of the West Indies, Mandeville Regional Hospital, May Pen Hospital and Spanish Town Hospital, all providing services for children and adolescents aged 0 to 15 years. The UHWI and Bustamante Hospital for Children accepted referrals island-wide for further care, including intensive care services. Hospitalized children and adolescents aged 0 to 15 years with pediatrician-diagnosed and/or laboratory-confirmed dengue were included. Cases were ascertained from the ward admission registers and MoHW Class 1 Notifiable Disease registers of all hospitals. A data extraction sheet was used to collect data after review of their hospital files. We also collaborated with the national surveillance records for dengue.

Samples at the UHWI were tested for dengue NS1 antigen using enzyme-linked immunosorbent assay (ELISA). While samples sent to the private laboratories were tested using rapid dengue NS1 antigen, dengue IgM and IgG serology. A subset of samples was tested using reverse transcriptase polymerase chain reaction (RT-PCR) at the UHWI and the Caribbean Public Health Agency (CARPHA) laboratories with serotypes identified. CARPHA also tested a subset for acute ZIKV and CHIKV infections by RT-PCR. Hematologic and biochemical tests were performed on blood samples routinely collected during hospital care.

Primary data collected included demographics, hospitalization, intensive care unit (ICU) admission, anthropometry, exposure to ill-contacts, previous arbovirus infections, co-morbidities, drug history, clinical presentation, complications, laboratory investigations including dengue RT-PCR and NS1 antigen, ZIKV infections (IgG), ZIKV and CHIKV RT-PCR, hematology, chemistry, radiology, treatment modalities including transfusion of blood products, organ-system involvement and outcomes.

Case definitions for “dengue” from WHO's revised criteria in 2009 were included: dengue without warning signs, dengue with warning signs, severe dengue without hemorrhagic features and severe dengue with hemorrhagic features. Cases of “pediatrician-diagnosed” dengue fever in hospitalized children were included. Laboratory-confirmed dengue was diagnosed by a positive dengue NS1 antigen and/or dengue RT-PCR. Children were managed according to PAHO criteria which were modified and national training done for their appropriate implementation and use (21).

Data were extracted from questionnaires, unlinked to patient identifiers, entered into a dataset on Microsoft Excel version 2016 and then exported to Statistical Package for the Social Sciences (SPSS) version 20 and analyzed. Comparisons were made between groups using Student's *t*-test for normally distributed parametric data. Chi-square analysis, or Fisher's exact test was used for categorical variables. Continuous variables were analyzed using Student's *t*-test. A *p-*value < 0.05 was considered statistically significant.

Ethics approval was formally obtained to perform this study from the Medical Research Ethics Committee of the University Hospital of the West Indies Mona Campus and also the Ethics **(ECP 218 18/19; September 16, 2019)** and also the Medico-legal Affairs Committee of the Ministry of Health and Wellness, Jamaica **(2019/18; November 11, 2019)**. Dengue fever is a Class 1 Notifiable Disease in Jamaica. Parents were informed of their child's diagnosis and its mandatory notification to the National Health Authorities within 24 h. Only medical records were extracted and there was no study intervention other than routine medical care, so there was no need for informed consent to be obtained from the parents.

## Results

A total of 339 children and adolescents fitting the criteria for dengue were enrolled. Of this total, 279 had testing for dengue NS1 antigen and/ or dengue RT-PCR. Of these, 220 (78.9%) had a positive laboratory confirmation: 218 (71.8%) were dengue NS1 antigen positive and 23 (6.8%) were RT-PCR positive, with overlap of 21 (7.5%), both dengue RT-PCR and NS1 antigen positive. DENV-3 serotype was confirmed in a subset of 20. No other arbovirus was co-circulating. Cases were reported from the Bustamante Hospital for Children (159), UHWI (130), Mandeville Regional Hospital (19), May Pen Hospital (16) and Spanish Town Hospital (15).

Aggregate results for 339 children were compared against the subset of 17 (5%) who died (Tables 1–**5**). Dengue classification, overall, comprized “severe dengue” in 68 (20.1%), those without hemorrhage in 28 (8.3%) and those with hemorrhage in 40 (11.8%). Among the 68 with severe dengue, more than half, 59% had hemorrhage. “Dengue with warning signs” were 218 (64.3%) and “dengue without warning signs” in 53 (15.6%). The children were aged 4 months to 15 years with a mean age 6 years. The majority of those affected were 6–10 years-126 (37.2%), followed by 1–5 years-119 (35.1%) (Table 1). The male:female ratio was 1:1.

**Table 1:** Demographics and co-morbidities in pediatric patients hospitalized with suspected and confirmed dengue in the 2018–2019 epidemic.

	**Dengue without warning signs** **(*n* = 53)**	**Dengue with warning signs** **(*n* = 218)**	**Severe dengue** **without haemorrhagic features (*n* = 28)**	**Severe dengue with haemorrhagic features (*n* = 40)**	**Total** **(*n* = 339)**	**Mortality cases (*n* = 17)**
**Age group**	**Number (%)**	**Number (%)**	**Number (%)**	**Number (%)**	**Total (%)**	**Number (%)**
<1 year	7 (2.1)	24 (7.1)	4 (1.2)	3 (0.9)	**38 (11.2)**	1 (5.9)
1–5 years	20 (5.9)	69 (20.4)	13 (3.8)	17 (5.0)	**119 (35.1)**	9 (52.9)
6–10 years	14 (4.1)	91 (26.8)	7 (2.1)	14 (4.1)	**126 (37.2)**	6 (35.3)
11–15 years	10 (2.9)	33 (9.7)	4 (1.2)	6 (1.8)	**53 (15.6)**	1 (5.9)
Missing data	2 (0.6)	1 (0.3)	0	0	**3 (0.9)**	0
**Sex**	**Number (%)**	**Number (%)**	**Number (%)**	**Number (%)**	**Total (%)**	**Number (%)**
Male	29 (8.6)	108 (31.9)	15 (4.4)	16 (4.7)	**168 (49.6)**	5 (29.4)
Female	22 (6.5)	109 (32.2)	13 (3.8)	23 (6.8)	**167 (49.3)**	12 (70.6)
Missing data	2 (0.6)	1 (0.3)	0	1 (0.3)	**4 (1.2)**	0
**Co-morbid illnesses**						
Hb SC	1 (0.3)	3 (0.9)	2 (0.6)	4 (1.2)*	**10 (2.9)**	3 (17.6)
Hb SS	0	6 (1.8)	2 (0.6)	0	**8 (2.4)**	0
Hb SBthal	0	0	0	1 (0.3)	**1 (0.3)**	0
Hb AS	1 (0.3)	4 (1.2)	0	0	**5 (1.5)**	0
Asthma	5 (1.5)	35 (10.3)	1 (0.3)	5 (1.5)	**46 (13.6)**	4 (23.5)
Cardiac	1 (0.3)	3 (0.9)	0	1 (0.3)	**5 (1.5)**	0
Epilepsy	0	4 (1.2)	2 (0.6)	2 (0.6)	**8 (2.4)**	2 (11.8)
Cerebral palsy	0	1 (0.3)	0	0	**1 (0.3)**	1 (5.9)
Others:	4 (1.2)	8 (2.4)	2 (0.6)	1 (0.3)	**15 (4.4)**	1 (5.9)

*p <0.05.

Co-morbidities were present in 88 (26%). The most commonly reported were: asthma 46 (52.3%), sickle cell disease 19 (21.6%) specifically Hb SC in 10 (11.4%) and epilepsy 8 (9.1%) (Table 1). Hb SC was associated with severe dengue with hemorrhage (*p* = 0.005). Non-steroidal anti-inflammatory drug use was reported in 36 (10.6%).

The time between onset of illness and hospitalization was 0–21 days (Table 2). There were 13 (3.8%) ICU admissions, all had severe dengue with hemorrhage of which 7 (53.8%) died. Duration of ICU admissions and hospitalizations ranged from 1 to 26 days and 1–46 days, respectively. Those with severe dengue with hemorrhage had the longest hospitalization (Table 2).

**Table 2:** Time of presentation and duration of hospitalization in pediatric patients hospitalized with suspected and confirmed dengue in the 2018–2019 epidemic.

	**Dengue without warning signs** **(*n* = 53)**	**Dengue with warning signs** **(*n* = 218)**	**Severe dengue** **without haemorrhagic features (*n* = 28)**	**Severe Dengue with haemorrhagic features (*n* = 40)**	**Mortality cases (*n* = 17)**
**Time between onset and presentation to hospital**	**Days**	**Days**	**Days**	**Days**	**Days**
Minimum-maximum	0–14	0–21	0–14	0–21	1–7
Median	4	4	4	3	3
Mean	4.44	4.67	4.43	4.08	3.12
CI (95%)	3.493–5.391	4.195–5.136	3.051–5.806	2.913–5.237	2.267–3.968
Duration of hospitalization	**Days**	**Days**	**Days**	**Days**	**Days**
Minimum-maximum	1–14	3–21	4–14	3–46	
Median	4	6	9	8	
Mean	5.07	6.06	8.63	12.04	
CI (95%)	4.35–5.80	5.72–6.39	7.46–9.80	7.64–16.44	

Three hundred and thirty-six (99.1%) had fever (Table 3A). Organ-system involvement occurred in 334 subjects (98.5%) (Table 4). More organ-systems were involved with increasing disease severity, with 39 (11.5%) having 5–7 organ-systems involved. The gastrointestinal system was most commonly affected in 317 (93.5%) (Table 4), including vomiting-223 (65.8%), abdominal pain/ loss of appetite-(53%), diarrhea-123 (36.3%) and hepatomegaly only-70 (20.6%) (Table 3A). Hematologic involvement occurred in 311 (91.7%) and musculoskeletal in 180 (53.1%) (Table 4). Other frequent clinical presentations included headache (54.9%), lethargy (53%), cough (34.5%), rash (28.9%) and eye pain (22.7%). Those who died had higher rates of lethargy (82.4%), loss of appetite and diarrhea (76.5%), vomiting (70.6%), hypotension (64.7%) and shock (58.8%) (Tables 3A–D).

**Table 3A:** Clinical manifestations in pediatric patients hospitalized with suspected and confirmed dengue in the 2018-2019 epidemic.

	**Dengue without warning signs** **(*n* = 53)**	**Dengue with warning signs** **(*n* = 218)**	**Severe dengue** **without haemorrhagic features (*n* = 28)**	**Severe dengue with haemorrhagic features (*n* = 40)**	**Total** **(*n* = 339)**	**Mortality cases (*n* = 17)**
	**Number (%)**	**Number (%)**	**Number (%)**	**Number (%)**	**Total (%)**	**Number (%)**
Fever	51 (15.0)	217 (64.0)	28 (8.3)	40 (11.8)	336 (99.1)	17 (100)
**Height of fever (** **°** **C)**
Minimum-maximum	38–40.6	38–41.3	38–42.1	38–40.2	**-**	38.0–39.7
Median	38.9	39	38.95	39.05	**-**	39.15
Mean	38.98	39.13	39.13	39.09	**-**	39.03
CI (95%)	38.72–39.24	39.01–39.25	38.69–39.58	38.83–39.34	**-**	38.66–39.40
Duration of fever (days)	**Days**	**Days**	**Days**	**Days**	**Days**	**Days**
Minimum-maximum	1–14	1–38	1–14	1–12	**-**	1–7
Median	4.5	4	5	4	**-**	3.5
Mean	4.43	5.03	5.5	5	**-**	3.5
CI (95%)	3.47–5.39	4.36–5.71	4.01–6.99	3.91–6.09	**-**	2.23–4.77
Hypothermia	0	0	1 (0.3)	5 (1.5)	**6 (1.8)**	**6 (35.3)**
Gastro-intestinal	**Number (%)**	**Number (%)**	**Number (%)**	**Number (%)**	**Total (%)**	**Number (%)**
Vomiting	30 (8.8)	145 (42.8)	17 (5.0)	31(9.1)	**223 (65.8)**	12 (70.6)
Diarrhea	15 (4.4)	76 (22.4)	9 (2.7)	23 (6.8)	**123 (36.3)**	13 (76.5)
Abdominal pain	10 (2.9)	124 (36.6)	21 (6.2)	27 (8.0)	**182 (53.7)**	10 (58.8)
Abdominal tenderness	4 (1.2)	86 (25.4)	21 (6.2)	23(6.8)	**134 (39.5)**	7 (41.2)
Hepatomegaly	0	38 (11.2)	12 (3.5)	20 (5.9)	**70 (20.6)**	10 (58.8)
Splenomegaly	0	2 (0.6)	0	1 (0.3)	**3 (0.9)**	1 (5.9)
Hepato-splenomegaly	0	4 (1.2)	4 (1.2)	2 (0.6)	**10 (2.9)**	1 (5.9)
Ascites	0	7 (2.1)	15 (4.4)	15 (4.4)	**37 (10.9)**	7 (41.2)

**Table 3B:** Clinical manifestations in pediatric patients hospitalized with suspected and confirmed dengue in the 2018–2019 epidemic.

	**Dengue without warning signs** **(*n* = 53)**	**Dengue with warning signs** **(*n* = 218)**	**Severe dengue** **without haemorrhagic features (*n* = 28)**	**Severe Dengue with haemorrhagic features (*n* = 40)**	**Total** **(*n* = 339)**	**Mortality cases (*n* = 17)**
**Musculoskeletal**	**Number (%)**	**Number (%)**	**Number (%)**	**Number (%)**	**Total (%)**	**Number (%)**
Arthralgia	20 (5.9)	65 (19.2)	13 (3.8)	15 (4.4)	**113 (33.3)**	4 (23.5)
Myalgia	9 (2.7)	47 (13.9)	5 (1.5)	9 (2.7)	**70 (20.6)**	2 (11.8)
**Skin**
Rash	19 (5.6)	61 (18.0)	7 (2.1)	11(3.2)	**98 (28.9)**	2 (11.8)
Petechiae	2 (0.6)	29 (8.6)	8 (2.4)	16 (4.7)	**55 (16.2)**	6 (35.3)
Ecchymoses	0	7 (2.1)	4 (1.2)	10 (2.9)	**21(6.2)**	5 (29.4)
Tourniquet test done	1 (0.3)	11(3.2)	3 (0.9)	0	**15 (4.4)**	0
Positive tourniquet test	1 (0.3)	3 (0.9)	3 (0.9)	0	**7 (2.1)**	0
Bleeding from lips/ mouth	0	3 (0.9)	0	2 (0.6)	**5 (1.5)**	0
**Cardiovascular system**						
Documented shock	0	0	6 (1.8)	13 (3.8)	**19 (5.6)**	10 (58.8)
Narrow pulse pressure	0	1 (0.3)	0	4 (1.2)	**5 (1.5)**	3 (17.6)
Hypotension	0	42 (12.4)	15 (4.4)	21 (6.2)	**78 (23)**	11 (64.7)
Postural changes	0	6 (1.8)	9 (2.7)	3 (0.9)	**18 (5.3)**	1 (5.9)
Pericardial effusion	0	1 (0.3)	0	4 (1.2)	**5 (1.5)**	3 (17.6)
Myocarditis	0	2 (0.6)	0	4 (1.2)	**6 (1.8)**	2 (11.8)
Oedema	1 (0.3)	25 (7.4)	16 (4.7)	14 (4.1)	**56 (16.5)**	4 (23.5)
Palpitation	0	7 (2.1)	0	3 (0.9)	**10 (2.9)**	0

**Table 3C:** Clinical manifestations in pediatric patients hospitalized with suspected and confirmed dengue in the 2018–2019 epidemic.

	**Dengue without warning signs** **(*n* = 53)**	**Dengue with warning signs** **(*n* = 218)**	**Severe dengue** **without haemorrhagic features (*n* = 28)**	**Severe dengue with haemorrhagic features (*n* = 40)**	**Total** **(*n* = 339)**	**Mortality cases (*n* = 17)**
**Respiratory/ Ear**, **Nose and Throat (for ENT)**	**Number (%)**	**Number (%)**	**Number (%)**	**Number (%)**	**Total (%)**	**Number (%)**
Cough	11 (3.2)	76 (22.4)	10 (2.9)	20 (5.9)	**117 (34.5)**	8 (47.1)
Post-tussive vomiting	0	5 (1.5)	0	0	**5 (1.5)**	0
Chest Pain	1 (0.3)	11 (3.2)	2 (0.6)	5 (1.5)	**19 (5.6)**	2 (11.8)
Wheeze	0	6 (1.8)	1 (0.3)	1 (0.3)	**8 (2.4)**	0
Pleural effusion	0	5 (1.5)	17 (5.0)	17 (5.0)	**39 (11.5)**	8 (47.1)
Ear pain	2 (0.6)	4 (1.2)	0	2 (0.6)	**8 (2.4)**	1 (5.9)
Runny nose	7 (2.1)	41 (12.1)	4 (1.2)	10 (2.9)	**62 (18.3)**	5 (29.4)
Stuffy	4 (1.2)	23 (6.8)	4 (1.2)	5 (1.5)	**36 (10.6)**	2 (11.8)
Sore throat	4 (1.2)	16 (4.7)	2 (0.6)	3 (0.9)	**25 (7.4)**	2 (11.8)
**Central nervous system (for CNS)**
Headache	25 (7.4)	124 (36.6)	15 (4.4)	22 (6.5)	**186 (54.9)**	0
Confusional state	0	6 (1.8)	0	2 (0.6)	**8 (2.4)**	2 (11.8)
Seizure	2 (0.6)	4 (1.2)	2 (0.6)	6 (1.8)	**14 (4.1)**	3 (17.6)
Seizure with fever	2 (0.6)	4 (1.2)	0	3 (0.9)	**9 (2.7)**	2 (11.8)
Complex febrile seizure	1 (0.3)	1 (0.3)	0	2 (0.6)	**4 (1.2)**	1 (5.9)
Syncope	0	6 (1.8)	1 (0.3)	2 (0.6)	**9 (2.7)**	2 (11.8)
Encephalitis	0	0	1 (0.3)	7 (2.1)	**8 (2.4)**	3 (17.6)
Encephalopathy	0	0	1 (0.3)	9 (2.7)	**10 (2.9)**	6 (35.3)
Phonophobia	0	9 (2.7)	2 (0.6)	2 (0.6)	**13 (3.8)**	0
Photophobia	1 (0.3)	8 (2.4)	0	2 (0.6)	**11 (3.2)**	0

**Table 3D:** Clinical manifestations in pediatric patients hospitalized with suspected and confirmed dengue in the 2018–2019 epidemic.

	**Dengue without warning signs (*n* = 53)**	**Dengue with warning signs (*n* = 218)**	**Severe dengue without haemorrhagic features (*n* = 28)**	**Severe dengue with haemorrhagic features (*n* = 40)**	**Total (*n* = 339)**	**Mortality cases (*n* = 17)**
**Eye**	**Number (%)**	**Number (%)**	**Number (%)**	**Number (%)**	**Total (%)**	**Number (%)**
Conjunctivitis	4 (1.2)	24 (7.1)	0	9 (2.7)	**37 (10.9)**	3 (17.6)
Pain	10 (2.9)	48 (14.2)	8 (2.4)	11 (3.2)	**77 (22.7)**	2 (11.8)
Sub-conjunctival hemorrhage	0	2 (0.6)	0	3 (0.9)	**5 (1.5)**	0
**Genitourinary (for GU)**
Increased urinary frequency	0	6 (1.8)	0	2 (0.6)	**8 (2.4)**	1 (5.9)
Decreased urine output	1 (0.3)	6 (1.8)	3 (0.9)	2 (0.6)	**12 (3.5)**	1 (5.9)
**Other symptoms**
Lethargy/ decreased activity	0	137 (40.4)	17 (5.0)	26 (7.7)	**180 (53.1)**	14 (82.4)
Restless	0	5 (1.5)	3 (0.9)	2 (0.6)	**10 (2.9)**	2 (11.8)
Irritable	10 (2.9)	30 (8.8)	6 (1.8)	9 (2.7)	**55 (16.2)**	3 (17.6)
Weight loss	1 (0.3)	9 (2.7)	3 (0.9)	1 (0.3)	**14 (4.1)**	1 (5.9)
Decreased/ loss of appetite	15 (4.4)	124 (36.6)	16 (4.7)	25 (7.4)	**180 (53.1)**	13 (76.5)
Malaise	0	2 (0.6)	0	3 (0.9)	**5 (1.5)**	1 (5.9)
Chills	4 (1.2)	9 (2.7)	2 (0.6)	3 (0.9)	**18 (5.3)**	0
Rigors	2 (0.6)	8 (2.4)	2 (0.6)	2 (0.6)	**14 (4.1)**	0
Fatigue	1 (0.3)	6 (1.8)	1 (0.3)	3 (0.9)	**11 (3.2)**	1 (5.9)
Weakness	4 (1.2)	38 (11.2)	2 (0.6)	12 (3.5)	**56 (16.5)**	5 (29.4)
Dizziness	4 (1.2)	11 (3.2)	1 (0.3)	3 (0.9)	**19 (5.6)**	0

**Table 4:** Organ-system involvement in pediatric patients hospitalized with suspected dengue in 2018–2019 epidemic.

	**Dengue without warning signs** **(*n* = 53)**	**Dengue with warning signs** **(*n* = 218)**	**Severe dengue** **without haemorrhagic features (*n* = 28)**	**Severe Dengue with haemorrhagic features (*n* = 40)**	**Total** **(*n* = 339)**	**Mortality cases** **(*n* = 17)**
**Outcome**	**Number (%)**	**Number (%)**	**Number (%)**	**Number (%)**	**Total (%)**	**Number (%)**
Alive	53 (15.6)	217(64.0)	27 (8.0)	25 (7.4)	**322 (95)**	-
Dead	0	1 (0.3)	1 (0.3)	15 (4.4)	**17 (5.0)**	17 (100)
Organ-system involvement # of cases (%)	48 (14.2)	218 (64.3)	28 (8.3)	40 (11.8)	334 (98.5)	17 (100)
**# of organs involved**						
Range	0–4	1–5	2–6	2–7	**-**	3–7
Median	2	3	4	5	**-**	5
Mean	2	2.72	4.25	4.55	**-**	5.294
CI (95%)	1.71–2.29	2.61–2.82	3.89–4.61	4.05–5.05	**-**	4.73–5.86
**Type of organ-system involved**	**Number (%)**	**Number (%)**	**Number (%)**	**Number (%)**	**Total (%)**	**Number (%)**
Gastrointestinal	37 (10.9)	213 (62.8)	28 (8.3)	39 (11.5)	**317 (93.5)**	17 (100)
Haematologic	39 (11.5)	204 (60.2)	28 (8.3)	40 (11.8)	**311 (91.7)**	17 (100)
Musculoskeletal	26 (7.7)	108 (31.9)	19 (5.6)	27 (8.0)	**180 (53.1)**	12 (70.6)
Cardiac	0	44 (13.0)	19 (5.6)	24 (7.1)	**87 (25.7)**	13 (76.5)
Respiratory	0	5 (1.5)	18 (5.3)	18 (5.3)	**41 (12.1)**	9 (52.9)
Renal	1 (0.3)	15 (4.4)	5 (1.5)	20 (5.9)	**41 (12.1)**	14 (82.4)
Central nervous system	3 (0.9)	3 (0.9)	2 (0.6)	13 (3.8)	**21 (6.2)**	7 (41.2)

Thrombocytopenia was the most common hematologic manifestation affecting 283 (83.4%) and was severe in 125 (36.9%) with platelet count <50 x 10^9^/L (Tables 5A,B). Leukopenia occurred in 169 (49.9%) (Tables 5A,B). The majority of those who died had severe thrombocytopenia (76.5%) (*p* = 0.003), deranged partial thromboplastin time (PTT) (70.6%), deranged prothrombin time (PT) (58.8%) and higher hematocrit concentrations >45% (47.1%) (Tables 5A,B). Elevated aspartate aminotransferase (AST) occurred in 245 (72.3%), where 3-10 times the upper limit for age was statistically significant in those with dengue with warning signs (*p* = <0.001). Elevated alanine aminotransferase (ALT) was seen in 147 (43.4%), elevated lactate dehydrogenase (LDH) in 164 (48.4%) and elevated creatine phosphokinase (CPK) in 84 (24.8%) with levels as high as 121,560 u/L. Higher levels of biochemical markers were observed in severe disease and those who died (Tables 5B,C). Elevated LDH and CPK levels were significantly associated with severe dengue with hemorrhage, *p* values = <0.001 and 0.002, respectively. A subset of NS1 positive samples was assessed for serotyping at the UHWI and CARPHA with all (20/20) identified as DENV-3.

**Table 5A:** Laboratory investigations in pediatric patients hospitalized with suspected and confirmed dengue in the 2018–2019 epidemic.

	**Dengue without warning signs** **(*n* = 53)**	**Dengue with warning signs** **(*n* = 218)**	**Severe dengue without haemorrhagic features (*n* = 28)**	**Severe Dengue with haemorrhagic features (*n* = 40)**	**Total**	**Mortality cases (*n* = 17)**
**Haematocrit**	**Number (%)**	**Number (%)**	**Number (%)**	**Number (%)**	**Total (%)**	**Number (%)**
<30%	0	4 (1.2)	3 (0.9)	2 (0.6)	**9 (2.7)**	3 (17.6)
30–35%	7 (2.1)	28 (8.3)	6 (1.8)	5 (1.5)	**46 (13.6)**	1 (5.9)
35.1–40%	26 (7.7)	90 (26.5)	7 (2.1)	13 (3.8)	**136 (40.1)**	4 (23.5)
40.1–45%	11 (3.2)	69 (20.4)	8 (2.4)	7 (2.1)	**95 (28.0)**	1 (5.9)
>45%	1 (0.3)	16 (4.7)	4 (1.2)	10 (2.9)	**31 (9.1)**	8 (47.1)
Htc. ≥ 20% for age	2 (0.6)	6 (1.8)	4 (1.2)	7 (2.1)	**19 (5.6)**	5 (29.4)
**WBC (<4 cell/mm3)**
Leukopenia (# of cases)	22 (6.5)	124 (36.6)	14 (4.1)	9 (2.7)	169 (49.9)	3 (17.6)
Range	1.05–3.90	0.51–3.98	1.38–3.90	1.00–3.96	**-**	1.81–2.60
Median	2.54	2.68	3.5	2.6	**-**	1.90
Mean	2.58	2.57	3.22	2.66	**-**	2.10
CI (95%)	2.24–2.93	2.43–2.72	2.77–3.68	1.92–3.40	**-**	1.03–3.18
**Platelet (10** ^**9**^ **/L)**
Thrombocytopenia (# of cases)	34 (10.0)	187 (55.2)	26 (7.7)	36 (10.6)	283 (83.5)	15 (88.2)**
≤ 50	6 (1.8)	75 (22.1)	20 (5.9)	24 (7.1)	**125 (36.9)**	13 (76.5)
51–100	19 (5.6)	82 (24.2)	3 (0.9)	10 (2.9)	**114 (33.6)**	2 (11.8)
101–149	9 (2.7)	30 (8.8)	3 (0.9)	2 (0.6)	**44 (13.0)**	0
Range	15–141	3–147	6–139	8–136	**-**	8–74
Median	83	61	28	33.5	**-**	33
Mean	82.29	65.79	42.96	44.19	**-**	31.13
CI (95%)	71.02–93.57	60.69–70.90	27.75–58.17	33.66–54.73	**-**	20.79–41.48

**p <0.01. The highest haematocrit, lowest WBC and lowest platelet count during hospitalization were used.

**Table 5B:** Laboratory investigations in pediatric patients hospitalized with suspected and confirmed dengue in the 2018–2019 epidemic.

	**Dengue without warning signs (*n* = 53)**	**Dengue with warning signs (*n* = 218)**	**Severe dengue without haemorrhagic features (*n* = 28)**	**Severe dengue with haemorrhagic features (*n* = 40)**	**Total (*n* = 339)**	**Mortality cases (*n* = 17)**
**Coagulopathy (# of cases)**	**Number (%)**	**Number (%)**	**Number (%)**	**Number (%)**	**Total (%)**	**Number (%)**
Prolonged PT	0	2 (0.6)	1 (0.3)	11 (3.2)	**14 (4.1)**	10 (58.8)
Prolonged PTT	9 (2.7)	78 (23.0)	18 (5.3)	26 (7.7)	**131 (38.6)**	12 (70.6)
**Biochemical**	**Number (%)**	**Number (%)**	**Number (%)**	**Number (%)**	**Total (%)**	**Number (%)**
Elevated AST	**25 (7.4)**	**163 (48.1)**	**27 (8.0)**	**30 (8.8)**	**245 (72.3)**	**15 (88.2)**
(<3 xULN)	18 (5.3)	71 (20.9)	4 (1.2)	3 (0.9)	**96 (28.3)**	0
(3-10 xULN)	7 (2.1)	77 (22.7)	10 (2.9)	7 (2.1)	**101 (29.8)**	2 (11.8)
(>10 xULN)	0	15 (4.4)	13 (3.8)	20 (5.9)	**48 (14.2)**	13 (76.5)
Range (u/L)	75–181	56–773	133–5,525	112–18,703	**-**	222–18,703
Median (u/L)	154	222	1341.50	1118.50	**-**	2,387
Mean (u/L)	137.91	255.28	1,814	2887.69	**-**	3729.69
CI (95%)	113.26–162.56	222.98–287.57	939.77–2688.23	1026.02–4749.36	**-**	749.38–6710.01
	**Number (%)**	**Number (%)**	**Number (%)**	**Number (%)**	**Total (%)**	**Number (%)**
Elevated ALT	**11 (3.2)**	**89 (26.3)**	**20 (5.9)**	**27 (8.0)**	**147 (43.4)**	**14 (82.4)**
(<3 xULN)	10 (2.9)	48 (14.2)	4 (1.2)	6 (1.8)	**68 (20.1)**	1 (5.9)
(3-10 xULN)	1 (0.3)	39 (11.5)	4 (1.2)	8 (2.4)	**52 (15.3)**	3 (17.6)
(>10 xULN)	0	2 (0.6)	12 (3.5)	13 (3.8)	**27 (8.0)**	10 (58.8)
Range (u/L)	43–134	31–452	48–4,552	34–4,369	**-**	55–4,369
Median (u/L)	59	99	395	305	**-**	809
Mean (u/L)	67.27	124.84	790.05	810.19	**-**	956.46
CI (95%)	48.18–86.36	105.77–143.91	283.37–1296.73	356.10–1264.29	**-**	265.33–1647.60

**Table 5C:** Biochemical investigations in pediatric patients hospitalized with suspected and confirmed dengue in the 2018–2019 epidemic.

	**Dengue without warning signs (*n* = 53)**	**Dengue with warning signs (*n* = 218)**	**Severe dengue without haemorrhagic features (*n* = 28)**	**Severe dengue with haemorrhagic features (*n* = 40)**	**Total (*n* = 339)**	**Mortality cases (*n* = 17)**
Elevated LDH - # of cases (%)	15 (4.4)	106 (31.3)	18 (5.3)	25 (7.4)***	164 (48.4)	10 (58.8)
Range (u/L)	382–1,299	293–3,123	373–5090	419–11708	**-**	1,640–9,997
Median (u/L)	674	660.5	888.5	2162	**-**	4385.5
Mean	672.60	777.64	1843.61	3345.04	**-**	5045.10
CI (95%)	543.71–801.49	695.38–859.90	1038.38–2648.84	2021.50–4668.58		2911.30–7178.90
Elevated CPK - # of cases (%)	7 (2.1)	49 (14.5)	10 (2.9)	18 (5.3)**	84 (24.8)	10 (58.8)
Range (u/L)	226–1,601	202–10,248	243–4,113	305–121560	**-**	343–63,211
Median (u/L)	303	449	1,031	1360.50	**-**	1360.50
Mean (u/L)	519.14	1279.71	1,297	13255.56	**-**	7535.90
Elevated GGT - # of cases (%)	1 (0.3)	44 (13.0)	13 (3.8)	20 (5.9)	78 (23)	10 (58.8)
Range (u/L)		31–598	41–861	34–515	**-**	34–515
Median (u/L)		64.5	141	87.50	**-**	78.50
Mean (u/L)		98.71	183.62	146.5	**-**	164.40
CI (95%)		66.47–130.94	53.13–314.10	74.41–218.59		40.81–287.99
Other:	Number (%)	Number (%)	Number (%)	Number (%)	Total (%)	Number (%)
Hyponatramia (<135 mmol/l)	2 (0.6)	23 (6.8)	4 (1.2)	10 (2.9)	**39 (11.5)**	6 (35.3)
Hypernatraemia (>145 mmol/L)	5 (1.5)	26 (7.7)	11 (3.2)	8 (2.4)	**50 (14.7)**	6 (35.3)
Elevated urea	2 (0.6)	17 (5)	4 (1.2)	19 (5.6)	**42 (12.4)**	14 (82.4)
Elevated creatinine	0	3 (0.9)	1 (0.3)	10 (2.9)	**14 (4.1)**	10 (58.8)

**p <0.01; ***p <0.001.

Seventeen (5%) patients died, 16 (94.1%) with severe dengue, of which 15 (88.2%) had hemorrhage. The ages ranged from 7 months to 15 years, mean age 5 years, subset 1–5 years - 9 (52.9%). The male:female ratio was 1:2.4. Eight (47.1%) had co-morbidities: 4-asthma, 3-sickle cell disease Hb SC, 2-epilepsy and one with cerebral palsy (Table 1). All had hematologic and gastrointestinal manifestations with more organ-system involvement of 5–7 organs in 11 (64.7%). Eleven (64.7%) died within 24–48 h of presentation despite resuscitation and transfusion of blood products.

## Discussion

According to PAHO, in this “new dengue epidemic cycle in the Americas” in 2019, children under 15 years of age were predominantly affected, representing 64% of severe dengue cases in Guatemala and 58% of all confirmed deaths in Honduras (6). In this study from Jamaica, severe cases of dengue in hospitalized children approached 20.1% and CFR was 5% in 2018 to 2019. Of the 339 cases in this study, ages 1–10 years were most affected. Hb SC, elevated LDH and CPK were all statistically significant laboratory markers for severe dengue with hemorrhage. Of the 17 who died, the most affected included those aged 1–5 years, females more than males and those with more organ-system involvement. An alarming 64.7% died within 24 to 48 h of presentation.

This severity of illness and attributable-mortality in hospitalized children, synchronized with the national surveillance data for all reported cases in the 2018–2019 epidemic (16). The 2018 to 2019 dengue outbreak in Jamaica was more intense, atypical and severe as compared to previous epidemics, with 5,903 reported cases in 2012 and 10,411 cases in 2018–2019 after its maiden ZIKV epidemic in 2016 (16). No other arboviruses were circulating. Those under 15 years of age represented 41% of the total number of 10,411 reported cases and 42% of those who were hospitalized. The overall increase in the number of severe dengue cases in 2019 was also observed in Honduras (20).

There is a paucity of detailed reports describing the clinical severity of dengue infection in pediatric populations in the “new epidemic cycle” which occurred after the maiden ZIKV epidemic in the Americas (6, 22). Our study was first contrasted to others in ZIKV-naïve populations. The proportion of severe dengue cases in 2018–2019 in our study of Jamaican children (20.1%) was greater than the 10.5% reported from the UHWI, Jamaica in 2012 and 17.4% from Queen Elizabeth Hospital, Barbados in 2009 (18, 23). However, it was less than the 26% reported in Dominican Republic in 2008-2009 (24). Severe cases reported in the Caribbean region of the Americas were significantly less than the Asia-Pacific: Philippines 76.5% (2009–2010) and Indonesia 73% (2009–2013) (25, 26).

Jamaica observed an increase in the number of dengue-related deaths in children. Of the 86 dengue-related deaths that were reported nationally, 53 (61.6%) occurred in children under 15 years of age. Our CFR of 5% was higher than the 3.7% reported in hospitalized children from the UHWI in 2012, the 1.7% for the Queen Elizabeth Hospital in Barbados in 2009 and <1% mortality rates reported in the Philippines and Indonesia (19, 23, 25, 26). However, our child-mortality rate was similar to the 5.1% reported for children in Dominican Republic in 2008-2009 (24).

In our study, sickle cell disease Hb SC was significantly associated with severe dengue with hemorrhage (*p* = 0.005) and comprised 17.6% of those who died. In Jamaica, during 2010–2012, those with genotypes Hb SC, or SS had significantly higher dengue-attributable CFR of 12.5 vs. 0.4% in the general population (27). During 2005–2013, in the French Americas, children under 15 years with Hb SC had a higher proportion of severe disease (*p* < 0.001) and more deaths (*p* = 0.02) (28). Hemoglobin concentration at presentation and change in hemoglobin concentration from steady state were also independent predictors of mortality, other than SS genotype (27).

Hematologic and biochemical changes can assist in the classification of disease severity, identify complications and initiate management with the aim to decrease morbidity and mortality. A higher proportion of those with severe disease and deaths had hematocrits >40%, severe thrombocytopenia (platelets <50 x 10^9^/L) and deranged clotting indices. This is congruent with findings found in Sri Lankan children aged 5–12 years with dengue in 2013–2014 (29). The rise in the hematocrit was rapid and higher in those with dengue hemorrhagic fever who also had significantly lower platelet counts. WHO recognizes this as a sign of severe disease and need for aggressive treatment (21).

Higher levels of AST and ALT were observed in those with severe disease, particularly with hemorrhage and was an indicator for identifying severe disease. Those who died in our study, had severe hepatic transaminitis with high levels of AST (18,703 u/L) and ALT (4,369 u/L). These findings are supported by studies from Sri Lanka (2013–2014), Malaysia (2014–2015) and India (2014–2015) (29–31). Higher levels of LDH and CPK were both significantly associated with severe dengue with hemorrhage (*p* = <0.002). Our findings are congruent with Indian reports using CPK and LDH as biomarkers of disease severity (32). WHO reclassified dengue in 2009 to include: “dengue without warning signs,” “dengue with warning signs” and “severe dengue.” This classification to identify severe cases had a sensitivity of 59–98% and specificity 41–99% (33). This classification may require further revision as it lacks specific parameters that define the signs and measurable clinical/laboratory parameters. It can also be expanded to include laboratory investigations such as elevated LDH and CPK, as predictors of disease severity.

We identified DENV-3 as the sole serotype in those patients who were tested in this study. This is consistent with MoHW data indicating that this serotype was nearly the sole serotype (97%) circulating in Jamaica during 2018-2019 along with a few cases of DENV-2 (3%). A meta-analysis from the South East and non-South East Asia showed that primary DENV-3 infection and secondary infection with DENV-2, 3 and 4 increased the risk of severe dengue infections (34). During the 2016 ZIKV epidemic in Jamaica, DENV was co-circulating with ZIKV and was predominantly of the DENV-3 serotype (12, 13). Yet, severe disease and case fatality rates in DENV-infected Jamaican children during 2019 were much higher than in 2016, suggesting that secondary DENV infection after a primary ZIKV infection could have impacted disease severity.

Antibody-dependent enhancement (ADE) is characteristic of dengue and other flaviviruses (22, 35–37). Dengue and ZIKV viruses are flaviviruses whose envelope proteins share ~50% sequence homology (22, 37). In Nicaragua, a 12.1% probability of symptomatic DENV-2 infection in children with a previous ZIKV infection vs. 3.5% in flavivirus-naïve children, suggested ADE (22). A prior history of ZIKV infection was also associated with severe dengue in 5.4 vs. 0.7% in flavivirus-naïve children (22). Enhanced infection of heterologous DENV 1,3,4 serotypes and a live tetravalent vaccine are linked to ADE (22, 38, 39). The ZIKV and DENV ADE correlation is still being debated.

Laboratory-confirmation of dengue infection was present in 79% (220) of our cases. “Pediatrician-diagnosed” clinical cases in hospitalized children and adolescents were accepted in the remainder. Some who had “dengue without warning signs” were hospitalized, if pediatricians were unsure whether cases with fever would evolve into “severe dengue,” septicemia, meningitis, or other illnesses. Others were hospitalized over an abundance of caution, if family support was questionable for monitoring and supporting the child, should there be sudden deterioration and inability to return promptly for treatment and care, in the context of an evolving epidemic with increased attributable pediatric morbidity and mortality. The surveillance systems, protocols and training were similar across the five hospitals, the Childrens' Hospital and UHWI had 85% of cases with the highest burden of disease severity, as would be expected for these referral hospitals.

A new wave of dengue emerged after the ZIKV epidemic with increased disease severity and attributable-mortality in children and adolescents, as was observed in Jamaica. This occurred *in tandem* with the highest ever reported incidence and severity of epidemic dengue fever in Jamaica and the Americas and in the aftermath of the ZIKV “epidemic of international concern” in Latin America and the Caribbean. This unique increase in severe dengue in PAHO's “new epidemic cycle” may be an isolated event, may represent the peak in the cyclical nature of epidemics, or may become the new norm. It also raises the important point for continued surveillance for arboviruses in general as the ZIKV “epidemic of international concern” caught the entire Americas off-guard and then unexpectedly may have impacted dengue disease severity subsequently. Notwithstanding, recent statistical modeling has shown that since March 2020, the COVID-19 pandemic with the associated public health interventions and social disruptions, including closure of schools, has reduced dengue transmission by 0.72 million cases, or 44%, in 23 countries within Latin America and South East Asia, including Jamaica, which experienced a 95% decrease in reported dengue cases in 2020 (40).

## Conference Presentation

https://www.zikaconference.com/scientific-program. This was the first, oral platform abstracted presentation, by Dr. Aileen Lue, in the First Scientific Session of the “Third International Conference on ZIKA and *Aedes*-related Infections,” at the Metro Marriott Hotel, in Washington D.C., Feb 13-16, 2020. Abstract−8815. This was the subject of the doctoral thesis in support of Dr. Aileen Lue's Doctorate in Pediatrics degree, from the University of the West Indies.

## Data Availability Statement

The original contributions presented in the study are included in the article/supplementary materials, further inquiries can be directed to the corresponding author.

## Ethics Statement

The studies involving human participants were reviewed and approved by the Medical Research Ethics Committee of the University Hospital of the West Indies Mona Campus and also the Ethics (ECP 218 18/19; September 16, 2019) and also the Medico-legal Affairs Committee of the Ministry of Health and Wellness, Jamaica (2019/18; November 11, 2019). Written informed consent from the participants' legal guardian/next of kin was not required to participate in this study in accordance with the national legislation and the institutional requirements.

## Author Contributions

AL, M-AR-D, and CC conceptualized and designed the work. AL, CC, SK, JD-T, TJ-P, CP, CM, JA, and KW-K acquired the data. AL and GG-S managed the dataset, exported, and performed data analysis. AL and CC drafted and critically revised the work and continually provided intellectual input, all other co-authors contributed to this process throughout. All co-authors commented on the proposal, the work, data interpretation throughout, approved the final version submitted herein for consideration for publication, and agreed to be responsible for all aspects of the work reported herein.

## Funding

CC, JA, GG-S, and KW-K received honoraria from the European Union's Horizon 2020 Research and Innovation Program under grant agreement 734857 to assist in implementing the study and/or to support international travel to research meetings to present this work. AL received a travel grant from the University of the West Indies, Post Graduate Studies, Mona Campus, Jamaica, in partial fulfillment of her Doctorate in Pediatrics degree to enable AL's international travel to present this work at an international arbovirus conference (as follows). The European Union and the University of the West Indies' Graduate Studies Department had no role in the design and/or conduct of the study. The works reported herein are entirely the responsibility of the coauthors.

## Conflict of Interest

The authors declare that the research was conducted in the absence of any commercial or financial relationships that could be construed as a potential conflict of interest.

## Publisher's Note

All claims expressed in this article are solely those of the authors and do not necessarily represent those of their affiliated organizations, or those of the publisher, the editors and the reviewers. Any product that may be evaluated in this article, or claim that may be made by its manufacturer, is not guaranteed or endorsed by the publisher.

## Abbreviations

ADE: Antibody-dependent enhancement
ALT: Alanine aminotransferase
AST: Aspartate aminotransferase
CARPHA: Caribbean Public Health Agency
CFR: Case fatality rate
CPK: Creatine phosphokinase
DENV: Dengue virus
DHF: Dengue hemorrhagic fever
ELISA: Enzyme-linked immunosorbent assay
ICU: Intensive Care Unit
IgG: Immunoglobulin G
IgM: Immunoglobulin M
LDH: Lactate dehydrogenase
MoHW: Ministry of Health and Wellness
NS1: Non-structural protein 1
PAHO: Pan American Health Organization
PCR: Polymerase chain reaction
PT: Prothrombin time
PTT: Partial thromboplastin time
UHWI: University Hospital of the West Indies
WHO: World Health Organization
ZIKV: Zika virus.
